# Single Nucleotide Polymorphism Markers with Applications in Conservation and Exploitation of Aquatic Natural Populations

**DOI:** 10.3390/ani13061089

**Published:** 2023-03-18

**Authors:** Roman Wenne

**Affiliations:** Institute of Oceanology, Polish Academy of Sciences, Powstańców Warszawy 55, 81-712 Sopot, Poland; rwenne@iopan.pl

**Keywords:** biodiversity, conservation genetics, fisheries, molecular population genetics, genomics, SNP, selection, aquatic organisms

## Abstract

**Simple Summary:**

In recent decades, societies, states and local authorities have become increasingly aware that for effective long-term management and protection of aquatic ecosystems and populations, it is necessary to take into account the genetic changes occurring in these populations. One type of high-resolution molecular marker suitable for studying the neutral and adaptive genetic diversity of populations is single nucleotide polymorphism (SNP). This review is an attempt to show the benefits of using SNPs to recognize natural populations of aquatic animals and detect the threats to them from accidentally or intentionally released farm animals, fishery and global climate changes. It is postulated that conservation actions should protect not only pristine natural populations that are endangered or overfished, but also protect populations of non-threatened species from unnecessarily released semi-domesticated animals. The enhancement of natural populations with farmed material usually reduces their genetic diversity. Experimental size-selective catches of artificially created populations have caused evolutionary changes in the life cycles of fishes. However, fishery-induced evolution in natural populations is difficult to observe. The negative measurable effects on populations can be expected when the number of breeding individuals is reduced below 100, which occurs very rarely in the sea and more often in fragmented freshwater streams, ponds and seasonal rivers.

**Abstract:**

An increasing number of aquatic species have been studied for genetic polymorphism, which extends the knowledge on their natural populations. One type of high-resolution molecular marker suitable for studying the genetic diversity of large numbers of individuals is single nucleotide polymorphism (SNP). This review is an attempt to show the range of applications of SNPs in studies of natural populations of aquatic animals. In recent years, SNPs have been used in the genetic analysis of wild and enhanced fish and invertebrate populations in natural habitats, exploited migratory species in the oceans, migratory anadromous and freshwater fish and demersal species. SNPs have been used for the identification of species and their hybrids in natural environments, to study the genetic consequences of restocking for conservation purposes and the negative effects on natural populations of fish accidentally escaping from culture. SNPs are very useful for identifying genomic regions correlated with phenotypic variants relevant for wildlife protection, management and aquaculture. Experimental size-selective catches of populations created in tanks have caused evolutionary changes in life cycles of fishes. The research results have been discussed to clarify whether the fish populations in natural conditions can undergo changes due to selective harvesting targeting the fastest-growing fishes.

## 1. Introduction

In recent decades, administrations and societies have become increasingly aware that for the successful long-term management and conservation of aquatic ecosystems and animal populations, it is essential to take into account the genetic changes that occur in these populations. As a consequence, the number of publications on the population genetics and genomics of aquatic species increases from year to year. The effectiveness of these studies has been significantly increased by the use of high-resolution molecular markers, such as microsatellites and, more recently, SNPs. The development of high-throughput genotyping techniques reduces the cost of population studies and increases their availability in countries around the world.

The intensive exploitation of marine and freshwater resources requires the accompanying conservation of biodiversity at local and worldwide scales. Conservation is to maintain the threads of life across time and involves the ecosystem and a species flock or species perspectives [1]. Molecular genetic information on phylogeny, population genetics and genomics, and on quantitative traits, is needed for preserving biodiversity. Evolution has been traditionally understood as a long time process, lasting hundreds of thousands or millions of years. However, there is growing evidence that in natural (wild) environments there are cases of recent speciation under the ecological mechanisms leading to reproductive isolation or genetic processes in the presence of gene flow, resulting in a divergence at the level of morphology, as in the case of Midas cichlids [2]. Recent advances in sequencing techniques, including reduced representation sequencing (genome scans), resulted in gathering large data sets of molecular markers useful in the identification of the genetic variation in populations and genome regions under the influence of natural selection [3,4,5,6]. Single nucleotide polymorphisms (SNPs) open new possibilities of finding local adaptations in natural populations and plasticity in the face of climate changes, defining the management units for the purposes of conservation biology, the estimation of the effects on wild populations of releases or escapes of hatchery-reared fish and the evolutionary consequences of harvesting in the wild [7,8,9,10,11]. SNP loci can be found in coding or noncoding genome regions and are considered as non-neutral or neutral markers. Non-neutral markers (outliers) are influenced by selection and can have a higher discriminatory power in population studies in comparison with neutral markers [12].

The main concerns of conservation genetics are defining populations and closely related species, the characterization of their polymorphism and its changes over time, the identification of hybrids in wild populations, the estimation of effective population size, estimation of inbreeding, and understanding the local adaptations and genetic polymorphism at quantitative trait loci in populations. An understanding of the spatial structure of populations enables the delineation of evolutionary conservation units [13,14]. Evolutionary significant units are groups of individuals adapted to the local conditions and reproductively self-maintaining. Recently, conservation genetics research has been supported by conservation genomics based on large-scale sequencing or genome resequencing resulting in the identification and genotyping of a large number of SNPs [15,16,17]. In practice, however, conservation genomics may not be accessible in many countries because of the insufficient expertise of practitioners and limited financial resources [18].

**Figure 1:**
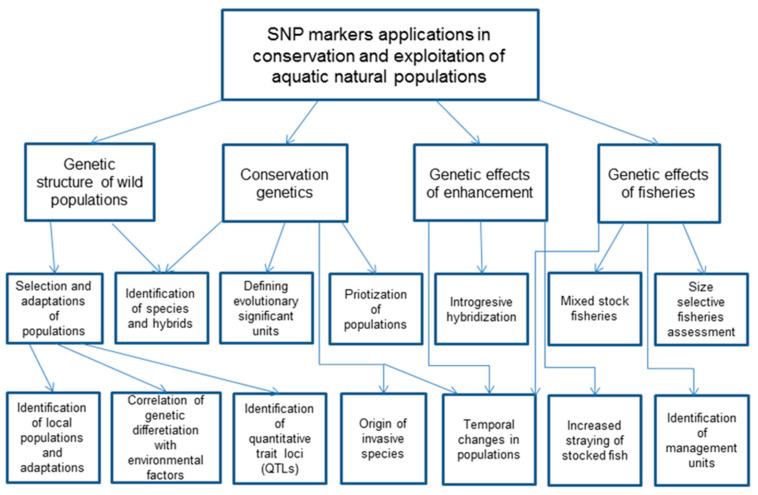
An integrated concept map showing the main fields and directions of SNP applications related to aquatic exploited animal populations.

There have been many review papers concerning the application of molecular markers to aquaculture and the management of fisheries’ resource conservation [6,7,13,19,20,21,22,23]. This review is an attempt to show the benefits of the application of SNPs to recognize natural populations of aquatic animals and to detect threats to them from accidentally or intentionally released farm animals (Figure 1).

Barcoding studies, although incomplete, have demonstrated the existence of a much larger number of species than expected. Using SNPs and next-generation sequencing (NGS) reveals more species and more fine-scale genetic differentiation over large geographic areas at an unprecedented scale, but also contributes to the discovery of the extent of the expansion of cryptic species and the invasions of species transported by shipping, byproducts of aquaculture introductions, natural expansions and climate changes. The SNP loci have been discovered and characterized for assays in specimens and populations of an increasing number of aquatic species [24]. The substantial number of successful applications of SNP markers in research on natural populations of fish and invertebrate species of interest to aquaculture and fisheries in most geographic areas has been reviewed. A list of some species is shown in Table 1.

## 2. Cross-Species Amplification of SNPs

Management and conservation activities require the correct identification of species and their hybrids in natural and hatchery environments. In some cases, sets of diagnostic SNPs have been constructed that enabled the simultaneous identification of a few species. A panel of SNPs was developed by cross-species amplification for Atlantic bluefin tuna, *Thunnus thynnus* and albacore, *T. alalunga* [96]. A set of 16 SNPs has been used for identification of 6 species of *Salvelinus* in lakes in Idaho, USA [97]. A panel of multiple diagnostic SNP loci was identified for the differentiation of three salmonid species using SNP-arrays [98]. The use of rainbow trout, *Oncorhynchus mykiss* and Atlantic salmon, *Salmo salar* sequence data was evaluated to identify SNPs in three other species: *O. tshawytscha*, *O. nerka* and *O. keta* [99]. The primers designed based on *O. mykiss* and *S. salar* alignments were more successful than the primers designed based on *Oncorhynchus*-only alignments of sequences. Approximately half of the subsequently designed validation assays resulted in high-throughput SNP genotyping markers. Smith et al. [99] suggest that this relatively low conversion rate may reflect the duplicated nature of the salmon genome. A suite of 12 subspecies and species-specific SNP markers was developed to distinguish the introduced rainbow trout, *Oncorhynchus mykiss* from the four major subspecies of cutthroat trout: westslope cutthroat trout (*Oncorhynchus clarki lewisi*), Yellowstone cutthroat trout (*Oncorhynchus c.i bouvieri*), coastal cutthroat trout (*Oncorhynchus c. clarki*), Lahontan cutthroat trout (*Oncorhynchus c. henshawi*), and their hybrids for stock assessments on the Clark Fork in Montana, USA [100]. A set of SNPs enabling discrimination of trout and their hybrids in *Salmo* genus, *Salmo marmoratus*, *S. obtusirostris* and different evolutionary lineages of *S. trutta* was designed [101]. Forty-one SNPs were species or lineage specific. This set of SNP markers may be applied as a molecular tool for genetic management purposes as well as in resolving the phylogenetic and taxonomic uncertainties of different trout stocks in southern Europe. Over 24,000 SNPs were successfully used for re-analysis of populations of two cryptic redfish species, *Sebastes mentella* and *S. fasciatus* in the North-West Atlantic (Canada), which will help to improve the assessment of fishery management units [102]. Restriction-site associated DNA (RAD) sequencing resulted in finding over 10,000 SNPs, which were used in population genomic studies of two African flatfish sibling species: *Sola senegalensis* and *S. aegyptiaca* [103]. The genomic research confirmed that they are genetically divergent but exchange genes asymmetrically across their natural hybrid zone near the Bizerte lagoon, with the shift of the cline into *S. aegyptiaca*. The identification of hybrids was more precise, with a large number of SNPs in comparison with earlier studies employing a low number of non-diagnostic markers. A large number of SNP markers useful for conservation purposes was discovered and characterized using RAD sequencing for four tetraploid sturgeon species, *A. naccarii* from the Adriatic Sea (hatchery), *A. gueldenstaedtii* and *A. persicus* from the Caspian Sea and *A. baerii* from Siberia [104]. High-throughput SNP-genotyping analysis among Ponto-Caspian sturgeon species from the Caspian Sea region revealed that Russian sturgeons (*Acipenser gueldenstaedtii*) from the Volga and Ural Rivers were essentially indistinguishable, but they differed from the Russian sturgeons in the Azov Sea, and from Persian (*A. persicus*) and Siberian river (*A. baerii*) sturgeons [105]. A species-specific SNP pattern in the nuclear MHC II antigen gene in museum samples of a now-extinct Baltic sturgeon population allowed for the detection of both European sturgeon *A. sturio* and American sea sturgeon *A. oxyrinchus* alleles [106]. The hybrid nature of the former Baltic sturgeon population should be taken into account in the current reintroduction measures. Short contig sequences (175,000) were obtained from a cultured GIFT (Genetically Improved Farmed Tilapia) strain of Nile tilapia (*Oreochromis niloticus*) and 3569 SNP were discovered [107]. In total, 384 SNPs were selected to monitor their applicability by genotyping the tilapia individuals from 3 species (*O. niloticus*, *O. aureus* and *O. mossambicus*), different strains and different geographical locations. Native Nile tilapia populations have been studied in areas where there is or will be aquaculture developed in order to trace the escapees or introductions from aquaculture, including the Volta river in western Africa. The introduced populations of Nile tilapia were studied in the Congo Basin using over 27,000RAD-derived SNPs [108]. The genetic differentiation of the introduced populations indicated independent introductions and hybridization with native species. The authors recommended the avoidance of further introductions and the incorporation of native species of tilapia into aquaculture. A 250,000-SNP array developed for the common carp *Cyptinus carpio* has been successfully used for genotyping eight other related species, including *C. carassius* and *D. rerio* [109]. Domestic and wild individuals were used for the construction of a 690,000 SNP arrays, allowing the genotyping of two species: channel catfish (*Ictalurus punctatus*) and blue catfish (*I. furcatus*) [110].

A SNP-based assay for identifying tanner crabs (*Chionoecetes bairdi*) from more commercial fishery targeted snow crabs (*C. opilio*) and their hybrids in the southeastern Bering Sea was developed [111]. The application of the type IIB endonuclease restriction-site associated DNA (2b-RAD) sequencing enabled finding a few thousand SNPs for East China deep sea mussel *Bathymodiolus species* (*B. platifrons*, *B. japonicus*, *B. aduloides* and *Idas* sp.), useful for both cross-species use and a fine-scale population differentiation assay [112]. Eight out of 38 Zhikong scallop (*Chlamys farreri*) SNP markers were also polymorphic in yesso scallop, *Patinopecten yessoensis* [113].

## 3. Genetic Structure of Wild Populations

SNPs have been useful in the analyses of the genetic structure of natural and wild populations of exploited species with high dispersal potential in ocean, anadromic, landlocked and sedentary freshwater populations and benthic species. Genetic differentiation between the pelagic populations of commercially important and over-fished albacore tuna, *Thunnus alalunga*, has been found on a large geographic scale in comparisons among the Pacific, Indian and Atlantic and Mediterranean Sea [96,114]. A differentiation between temperate and tropical groups of samples of swordfish (*Xiphias gladius*) was found in the Pacific, with heterogeneity detected among tropical samples [115. More pronounced differentiation was found between samples from the North and South Atlantic vs. Mediterranean spawning areas [116], and between Indian Ocean and South Atlantic populations [117]. Alternatively, the American eel (*Anguilla rostrata*) is a panmictic species, despite it inhabiting different environments from the southern Sargasso Sea to Canada and Greenland. The polygenic basis of discrimination between highly differentiated ecotypes rearing in brackish, saltwater and freshwater and was demonstrated experimentally [118]. Three hundred and thirty-one SNPs out of 42,424 were associated with 101 genes at co-varying loci. The genetic differences between ecotypes occur every generation anew.

The differences between west and east Atlantic cod *Gadus morhua* populations were found for the first time at the SNP allele frequencies in the transferrin gene [119]. Three hundred and eighteen segregating and twenty-nine outlier SNPs were discovered and the differences between North-East Arctic cod and Norwegian coastal cod were observed in their allele frequencies [120]. A genome scan analysis of 98 gene-associated SNPs revealed 8 outlier gene loci, likely to be subject to directional selection in local demes on a regional south/north transect of Central and Eastern Atlantic populations, with 7 loci displaying strongly elevated levels of genetic differentiation [121]. In addition, in the North Sea and Baltic Sea populations, four loci displayed evidence of adaptive evolution and were correlated with the temperature and/or salinity, evidencing selection acting in local populations. Over 1500 loci were genotyped in North Atlantic cod samples and a differentiation between 13 populations from North America and northern Europe was found [122]. Significant population differentiation among spawning groups of cod within the Gulf of Maine and between the Gulf of Maine and Georges Bank was found in three genomic linkage disequilibrium blocks (islands) using pairwise linkage analysis among 3,390,654 SNPs discovered by high-throughput sequencing [123]. The genomic blocks contained 1031 genes. In the studies of northeast Atlantic cod populations, a genomic region of strong population differentiation between the migratory and stationary ecotypes was identified [124]. The region extended over approximately 20 cM and suggested the involvement of a selective sweep. It is potentially associated with ecological divergence. Four overfished north-west populations of Atlantic cod in Canada, based on the otoliths collected during an 80-year period, were analyzed using over 1000 SNPs [125]. In total, 77 loci had highly elevated levels of differentiation. Temporal outliers in the different populations and outliers from the 1928 to 1960 period were stable in later decades. Temporal allele frequency shifts at certain loci might correlate with local temperature variation and with life history changes suggested to be fisheries-induced and driven by varying selection. The analysis of samples of historical otoliths of Arctic populations from Greenland and Iceland with a panel of over 900 SNPs revealed the existence of four genetically distinct groups that exhibited a considerable mixture [126]. The genetic composition had been stable over decades at some spawning groups, whereas the replacement of the population was recorded at others. Genetic differentiation between eastern (the Bay of Gdansk, Poland) and western (the Kiel Bight, Germany) Baltic populations existing in differentiated salinity conditions has been confirmed by the use of a cod-derived SNP array (Illumina) with 10,913 SNPs [127,128]. Discrete regions of divergence within the Atlantic cod genome are subject to directional selection and are associated with an adaptation to the local environmental conditions in the Baltic and the North Sea [129]. A set of outlier SNPs was located close to or within the genes associated with osmoregulation, and genes related to hydration and the development of oocytes. The adaptation of an eastern Baltic population to the low salinity water has been confirmed experimentally [130,131,132,133].

A much stronger differentiation has been revealed for migrating Atlantic salmon *Salmo salar* with the application of genome-wide SNP arrays [134]. Two main groups of European and North American populations and three major regional groups within Europe were observed. The neutral and outlier SNPs (putatively adaptive) with divergent allele frequencies across populations were found. The secondary contact zones were identified in Europe. Salmon SNP arrays can be applied for the conservation and management of populations. Highly informative SNPs can be used for cost-effective genetic stock identification [135]. A panel of 288 informative out of 5568 screened SNP markers was effectively used to examine both the individual assignments and mixed-stock fisheries of Atlantic salmon (*Salmo salar*) in Scotland and northeast England [136]. The baseline neutral *S. salar* SNP loci and loci determining sex and sea age at maturity SNP modules developed for the analysis of long-term genetic changes in the Teno River, Finland, were postulated to also be sufficient for the stock identification for other populations in Europe [137]. Ninety-six SNPs with the highest *F*_ST_ enabled the improvement of genetic stock identification of Chinook salmon (*Oncorhynchus tshawytscha*) from western Alaska in a study of twenty-eight populations [138]. The genetic differentiation of chum salmon *Oncorhynchus keta* populations found with SNPs had a regional structure in the northeastern Pacific Ocean, similar to that detected with microsatellites and allozymes [139]. The genetic variation was also found between the stocks differing by migration time. A panel of 89 SNPs was sufficient for mixed fishery analysis. On a broader geographic scale, throughout the entire range of chum salmon in North America and Asia, evidence of ascertainment bias, variable linkage relationships between SNPs associated with ancestral groupings and outlier loci with alleles associated with latitude were observed [140]. The analysis of 45 single nucleotide polymorphisms (SNPs) from 172 populations ranging from Russia to California of chinook salmon *Oncorhynchus tshawytscha* allowed for the grouping of populations into major lineages and mixed-stock analysis [141]. Eleven anadromous and resident populations of rainbow trout *Oncorhynchus mykiss* from the northwestern United States and British Columbia were divided into two major lineages by genotyping with a panel of 276 SNPs [142]. The evidence for divergent selection was found at a few candidate loci included a significant correlation with temperature. A signature of balancing selection was found in the major histocompatibility complex (MHC) class II (DAB) gene. Kokanee, the freshwater form of sockeye salmon (*Oncorhynchus nerka*), occur as two reproductive ecotypes with no morphological differences in British Columbia, Canada. Eighteen outlier SNP loci were identified as putative under the divergent selection between the ecotypes in the absence of a neutral SNP structure, indicating the early stages of ecological divergence [143]. Mixed-stock analyses of sockeye salmon from the Copper River, Alaska, revealed SNPs under diversifying selection, which increased the precision of the identification of the stock origin of individuals [12]. The differentiation between the spawning area sockeye ecotypes in the Wood River basin, Alaska (streams, rivers, lake beaches) at SNP loci possibly involved in local adaptations and was located in ‘genomic islands’, whereas neutral loci were spread throughout the genome [144]. A differentiation at the SNP loci in brown trout *Salmo trutta* native and supplemented populations has been reported in Europe [36,145,146,147].

Genetic differentiation, on European and local scales in the Atlantic, North Sea, northern Portugal, western, central and eastern Mediterranean have been found for populations of European hake, *Merluccius merluccius* [148,149], in particular with outlier loci. A clear distinction between the European anchovy *Engraulis encrasicolus* populations in the Atlantic and Mediterranean areas was observed and lower differentiation among Mediterranean populations [150]. Atlantic herring (*Clupea harengus*) populations are genetically structured at a fine scale within the Baltic Sea, which is explainable by oceanographic connectivity [151]. Whole-genome sequencing revealed ecological adaptations associated with the differences in allele frequencies at a large number of loci in populations of herring from a geographic range on both sides of the North Atlantic [152]. However, a panel of only 64 SNPs was sufficient to characterize the genetic structure of herring populations in the Gulf of St. Lawrence [153]. The application of a 60,000-SNP combined species arrays to population genetics has shown a slight differentiation between Iberian Atlantic and Mediterranean Sea bream (*Sparus aurata*) as well as between western and eastern Mediterranean European sea bass (*Dicentrarchus labrax*) [52]. A significant genetic differentiation of Asian seabass *Lates calcarifer* populations has been observed in Australia and Papua New Guinea, whereas lower differentiation has been seen in the Indonesian archipelago to Southeast Asia, with gene-associated SNP markers [154] and genetic polymorphism in the spotted scat, *Scatophagus argus* wild populations in China [155]. Eulachon, *Thaleichthys pacificus*, populations ranging from the Cook Inlet, Alaska, and along the west coast of North America to the Columbia River were analyzed [156]. The population structure assayed with the outlier SNPs provided a greater resolution of stocks and a higher individual assignment accuracy compared with a putatively neutral panel of 3911 SNPs and with 14 microsatellites. A cline of increasing genetic diversity from south to north was found in the adaptive SNP panel, but not in the neutral markers.

Four wild stocks of the giant freshwater prawn (*Macrobrachium rosenbergii*) in India were studied using high-throughput sequencing [157]. Out of 320 SNPs detected, 134 were common to all stocks and 3 were diagnostic. Four pathogen-defense genes were highly polymorphic. The Alaskan red king crab from 17 localities was studied in the North Pacific using 15 SNP loci [158]. Three geographically distinct evolutionary lineages were identified: Okhotsk Sea–Norton Sound–Aleutian Islands, southeastern Bering Sea–western Gulf of Alaska and Southeast Alaska. SNPs differentiating a Pacific mollusk abalone (*Haliotis discus hannai*) from China and Japan have been developed and can be used for the assessment of genetic structure changes in both wild and cultured populations [159]. SNPs were also used for assessment of spatio-temporal genetic variation in the South African abalone, *Haliotis midae* [160]. Temporal differentiation between samples can be explained by differential reproductive performance and changes in selection pressures over time. One hundred and three loci were amplified using pooled DNA templates from individuals of four wild Zhikong scallop, *Chlamys farreri*, populations in China: Changdao, Rongcheng, Rizhao and Qingdao [161]. The polymorphism assessment was performed in 60 individuals from the Rongcheng population, and 49 SNPs were polymorphic. The study of 1130 genome-wide SNP loci unraveled the effects of gene flow and the effects of selection on three populations of the silver-lip pearl oyster (*Pinctada maxima*) in the ecologically and economically important Indo-Pacific region: Aru, Bali and West Papua [162]. Twenty-two outlier SNPs were identified, giving evidence of strong local adaptation of populations, including two divergent groups (Bali/West Papua and Aru) despite the high gene flow. The genetic population structure of the black-lip pearl oyster *Pinctada margaritifera* was studied across its ~18,000 km Indo-Pacific distribution, using 580 specimens [163]. Analyses of 9624 genome-wide SNPs revealed 3 discrete genetic groups in the Indian and 5 in the Pacific Ocean. SNPs under directional selection did not show a higher number of groups in comparison with the neutral SNPs. The population’s genetic structure was coherent with the isolation by distance model and influenced by the ocean currents.

Northern and southern populations of blue mussels *Mytilus* differ in their genetic composition and belong to a few different taxa, which can hybridize [164]. Novel SNP markers were identified in European populations and contributed multilocus information on the distribution of three taxa: *M. trossulus*, *M. edulis* and *M. galloprovincialis* [165,166,167]. The SNP markers revealed largely concordant clinal variation across the hybrid zone between *M. edulis* and *M. trossulus* in the Baltic Sea [168]. The coexistence and hybridization of *M. trossulus* and *M. edulis* mussels in Greenland has been reported [169]. Invasive *M. galloprovincialis* threaten regional-scale genetic diversity in the mainland and remote offshore locations in the Southern Ocean [170,171,172]. This species recently invaded the Atlantic coasts of South America [173,174]. A significant differentiation of *M. chilensis* natural and cultured populations in the wild (Reloncavi, an inner bay influenced by aquaculture; Chiloe Island outer bay, and Patagonia, the most distinct population) was also delineated using neutral and outlier SNPs [175].

## 4. Genetic Effects of the Enhancement of Wild Populations with Hatchery-Reared Stocks

Salmonid populations have been strongly affected by introgressive hybridization caused by stocking with hatchery-reared fish over a long time. Nine populations of brook charr (*Salvelinus fontinalis*), either not stocked or stocked with a different intensity since 1971, were sampled in the Portneuf Wildlife Reserve in Quebec, Canada and genotyped at 280 SNPs [176]. The intensive stocking increased the genetic diversity, decreased the population differentiation and increased the individual admixture proportions. Twenty-seven SNPs including seven outliers, in a genomic cline analysis, showed an introgression rate either more restricted or enhanced relative to the neutral expectations. This indicated that hatchery selection mainly for growth has favored or hampered the introgression of genomic blocks into the wild populations, infringing the genomic integrity of wild populations and changing their functioning. The genetic impacts of gene flow caused by the straying of hatchery-released fish on wild populations of Atlantic salmon in the Gulf of Finland, in the Baltic Sea, over 16 years, 1996–2012, was assessed [177]. A panel of 1986 SNPs revealed that introgression changed the genetic composition of wild populations and increased the genetic diversity and lowered the genetic divergence. Introgression was higher in the eastern part and lower in the western part of Estonia, which may be related to the history of past stocking activities. The intensity of introgressive hybridization was differentiated in the genome of Atlantic salmon. The introgression most likely changes the functioning of indigenous populations.

SNP genotyping of historical fish scales, e.g., in sockeye salmon, *Oncorhynchus nerka* [178], and bones, e.g., on Pacific herring, *Clupea pallasi* [179], has been developed in the recent years. To investigate the genetic relationship between the extinct Atlantic salmon *Salmo salar* populations in Poland, southern Baltic Sea in 1950–1965 and the present-day populations, archival scale samples were studied using the Atlantic salmon Illumina 7000-SNP array [180,181]. Attempts to restitute the salmon populations in Poland have been based on a Latvian salmon population from the Daugava River. Releases of hatchery-reared smolts started in 1986, but to date, only one population with confirmed natural reproduction has been observed in the Slupia River. The historical salmon population from the Oder River, Poland, was genetically closer to present-day salmon from the Neman River, Lithuania, than to the historical salmon from the Vistula River, Poland and Dougava River, Latvia. Using geographically closer Neman River salmon instead of Dougava River, Latvia, for further restoration practices in Poland has been recommended. The recent genetic changes in populations of sea trout (*Salmo trutta* m. *trutta*) in the southern Baltic rivers in Poland in 1996–2009 related to enhancement practices were revealed with SNP analysis [182]. Two Soča River populations in Slovenia were analyzed with a set of 47 diagnostic SNP markers and consisted of more than 20% pure native Adriatic marble trout (*S. t. marmoratus*) individuals, a lineage of brown trout *S. trutta* [183]. Non-pure Danubian brown trout individuals were found in two Sava River hybrid populations and only seven contained fewer than 15% non-autochthonous Atlantic genes, suggesting the establishment of hybrid swarms. SNPs were used for detecting introgression and hybridization between subspecies of rainbow trout *Oncorhynchus mykiss*, called redband trout, in the upper McCloud River watershed in California [184]. Populations in only 4 creeks out of 16 studied contained a large portion of the non-introgressed redband trout individuals. The hybridization and introgression of introduced rainbow trout *O. mykiss* with native cutthroat trout *O. clarkii* populations in western North America due to widespread stocking and invasion into historical cutthroat trout habitats has been reported [185]. Genetic analysis, including 5 diagnostic autosomal SNPs, of the threatened Paiute cutthroat trout (*O. clarkii seleniris*) population, endemic to Silver King Creek, California, USA, revealed that it was weakly structured and introgressed from the long-time stocking by California golden trout (*O. mykiss aguabonita*) and hatchery rainbow trout *O. mykiss* [186]. The cutthroat trout proportion and effective population size of the admixed population were estimated. This will be useful in prioritizing the conservation and restoration programs for the introgressed populations. The extensive submersible mariculture of Atlantic salmon (*Salmo salar*) in hatcheries along the North Atlantic coasts has resulted in frequent escapes to the wild. It has been known that domestic salmon interbreed with wild populations and lower their fitness. The application of a 220,000-SNP array to studies of wild European and North American populations, farmed salmon and escapees confirmed and quantified the levels of European alleles and their introgression in Canada [32]. Subsequently, a large number of SNPs has been reduced to a 301 diagnostic set with a more than 95% fidelity of admixture identification [187].

Greenlip abalone (*Haliotis laevigata*) is an endemic Australian shellfish species targeted by fisheries and stock enhancement [188]. Thirteen locations along the Western Australian Greenlip Abalone Fisheries 8 Management Subareas were sampled for a population genetic survey [189]. A total of 69,720 SNPs were discovered, from which 18,803 SNPs were selected for subsequent analysis, including 1026 outliers. Genetic differentiation between samples from most locations was very weak for the neutral SNPs, suggesting the existence of a single population and isolation by distance. Contrary to this, the outliers (adaptive variation) suggested the existence of at least five differentiated groups, possibly related to the individuals adapted to an oxygen concentration range. No evidence for the reduction in genetic diversity related to overexploitation was found. For the purposes of stock enhancement, the broodstock should be taken directly from the genetic group of abalones to be enhanced, or from the most genetically similar group available [189].

## 5. Conservation Genetics

The first condition for the protection of natural populations and their proper management is the identification of species’ genetic characteristics of their populations. Molecular markers such as mtDNA, microsatellites and MHC turned out to be sensitive enough to find a population differentiation generally congruent with the genomic methods based on the genotyping of a large number of SNPs in the conservation units of coho salmon (*Oncorhynchus kisutch*) in Canada [14]. However, the genotyping of many thousands of SNPs has revealed a more detailed structure in some evolutionary and biologically significant units. Despite stocking being carried out with material from a well-defined management unit, enhancement leads to a reduction in the diversity of this population compared to other wild populations. This is due to the reduction in the effective population size and the constraints of the gene pool representation [14]. Nevertheless, fisheries’ overexploitation of many declining populations is mitigated by stocking. Negative effects of supportive breeding in wild populations appear when the number of broodfishcontributing to the stocking of natural populations is too low [190]. Broodfish should be replaced with individuals originating from wild populations. The success of enhancement depends on the spawning of families and the mortality of their progeny. Spawners and their progeny of red drum, *Sciaenops ocellatus*, were grown in three ponds and genotyped using a panel of 2000 SNPs [190]. It has been confirmed that the spawning events were dominated by a low number of usually younger spawners held for shorter periods in the hatchery, which caused a reduction in the genetic diversity in stocking material. A panel of 142 SNP loci was optimized for the parentage assignment and 35 SNPs for population assignment in the local creeks in Alaska for sockeye salmon, *Oncorhynchus nerka*, which allowed for their economic survey [191].

The scalloped hammerhead (*Sphyrna lewini*) is a critically endangered shark species characterized by a circum-global distribution in tropical and subtropical ocean zones. Genotyping with over 5000 SNPs confirmed the differentiation of Gulf of California samples from other regions of the Indo-Pacific, a lack of gene flow between populations from the Seychelles (Indian Ocean), Hawaii and Gulf of California and central Indo-Pacific locations and significant *F_ST_* among distant geographic regions [69]. On the basis of the obtained results, four main stocks have been delineated, including two in the Pacific. International collaboration is needed in order to implement conservation measures for this species. Genotyping with 59 diagnostic SNPs enabled mixed-stock analysis and the identification of management units of spring and autumn spawning herring, *Clupea harengus*, in the North and Baltic Seas [34].

Recent research encompassed 12 *Oreochromis* species from Tanzania, including native and introduced ones and hybrids, which are overall difficult to identify using phenotypic characters [192]. Whole-genome resequencing was applied to the analysis of 25 reference individuals representing four pure species and hybrids with introduced *O. niloticus* and *O. leucosticus*. The diagnostic panel of 96 SNPs for most of the 12 species studied and hybrids between 3 species (*O. urolepis*, *O. lecucostricus* and *O. niloticus*) was more diagnostic than the 18 microsatellites. This diagnostic panel of SNPs can be optimized further for identification of all 37 *Oreochromis* species and their hybrids. Such a panel of SNPs can be very useful in conservation activities directed at protection and restitution of native populations from hybridization and introgression of the introduced alien species originating from aquaculture.

SNPs are also useful in studies of the genetic structure of endemic species with local distribution only, such as Japanese lates (akame), *Lates japonica*. This freshwater species is distributed only in Kochi and Miyazaki areas in Japan, where two populations were identified using a set of 780 SNPs obtained from ddRAD-seq (restriction site—associated sequencing) [193]. In Tasmania, in the small Dervent Estuary, over 4000 SNPs enabled the identification of three main groups of populations of the spotted handfish (*Brachionichthys hirsutus*), which are recommended to be considered as separate conservation management units by the authors [194]. The threatened populations of subspecies of limpet *Patella candei* distributed only in the northeast Atlantic Macaronesian islands (e.g., the Canary Islands) have been identified with over 3000 SNP [195]. SNPs can be used for estimating the effective population size with a linkage disequilibrium method. Studies of thornback ray *Raja clavata* samples including 159 individuals from the Bay of Biscay [196] revealed that the precision of Ne estimates increases with the increase in the number of SNPs and stabilizes for approximately 1400 SNPs.

## 6. Selection and Adaptation of Aquatic Populations to Environmental Conditions

The use of a large number of genotyped SNPs makes it possible to identify the outlier loci and determine their correlation with environmental conditions, such as salinity, temperature, precipitation, food accessibility or migration distance and timing, and thus indicate possible adaptations [197]. However, the low effective population size, founder effect and population expansion can also affect the allele frequency at SNP loci.

Understanding the interactions between organisms and the environment requires the construction of genetic maps and the identification of quantitative trait loci (QTLs) related to candidate genes associated with the phenotypic traits influencing adaptations [198]. Most traits are usually complex and are controlled by a number of QTLs [23]. Nevertheless, natural populations are a source of genetic polymorphism including QTLs and their finding make it possible to build stocks that are more resistant and suitable for aquaculture. The identification of QTLs also benefits conservation efforts in the face of expected climate changes. SNPs are increasingly used for the identification of QTLs and can be located in the exons of candidate genes, including natural populations. Three QTLs for temperature tolerance and one for body size has been found in chinook salmon *Oncorhynchus tshawytscha* [198]. The application of SNP arrays and genome resequencing has uncovered significant genetic divergence between a pelagic shallow water morph of Arctic charr *Salvelinus alpinus* in Gander Lake in Newfoundland, Canada [199]. An outlier analysis revealed the divergence of functionally important genomic regions and a strong difference in the copy number of many hundreds of genes. One SNP (C > T) was associated with growth in the candidate gene TP53BP2 (tumor protein p53 binding protein 2) and some SNPs were potentially associated with head size and shape traits were found in bighead carp (*Hypophthalmichthys nobilis)* from the Yangtze River [200,201].

Of over 1600 SNPs genotyped in cod (*Gadus morhua*) populations, 40 outlier SNPs have been associated with the temperature in populations on both sides of the North Atlantic, which indicates the existence of discrete regions of genomes (linkage groups) under directional selection and may represent local adaptations [202]. The application of 12,000-SNP array to population studies of cod enabled the identification of four main chromosomal rearrangements associated with migratory behavior and environmental gradients (temperature, salinity and dissolved oxygen) across the North Atlantic species range [48,203]. Dozens of SNPs were found to be associated with environmental variables and most of them were identified in chromosomal rearrangements. Contrary to the non-migratory ecotype, the migratory ecotype possesses two inversions within the linkage group LG1 [204]. The rearranged regions have diverged in populations on both sides of the North Atlantic. Chromosomal rearrangements associated with migrations in rainbow trout (*O. mykiss*) also have been reported [205]. In herring *Clupea harengus* stocks differing in spawning time in North Atlantic, the genetic bases for adaptation were related to mutations in the *TSHR* gene and retrotransposon insertion [206,207]. A few outliers associated with the temperature and salinity have also been observed in the latter species [208]. In order to identify the genetic loci affecting the sea age at maturity in Atlantic salmon (*S. salar*), a genome-wide association study (GWAS) was performed using a pool-sequencing approach on fish from rivers and cultures in Norway [209]. The study revealed one major selective sweep, which covered 76 significant SNPs in which 74 were found in a 370 kb region of chromosome 25. Genotyping domesticated fish narrowed the haplotype region to four SNPs covering 2386 bp, containing the vgll3 gene, including two missense mutations explaining a 33–36% of the phenotypic variation. A single locus was found to have a highly significant role in shaping the sea age at maturation in this species. A survey of a few thousand SNPs in Mediterranean populations of sea bream *Diplodus sargus* and striped red mullet *Mullus surmuletus* revealed outlier SNPs correlated with temperature in the latter only [210]. The blue shark *Prionace glauca* was thought to have a panmictic world-wide population. The genome scan (RAD-DArT sequencing) enabled the discovery of over 37,000 SNPs [211]. Their genotyping showed a differentiation between the Northern (Mediterranean, northern Atlantic) and Southern populations (the Indo-West Pacific). The authors recommend the reconsideration of unit stocks for regional stock assessment of blue sharks, using the results of population genetic analysis.

A GWAS was used to find loci associated with resistance to rotten body disease caused by scuticociliates in large yellow croakers (*Larimichthys crocea*) [212]. A large number of fish from natural populations from China coastal waters have been analyzed by genome resequencing. Fourteen SNPs have been identified as associated with disease resistance, nine have been confirmed and five candidate genes have been found. Two SNPs have been associated with higher resistance to the reovirus and the mRNA expression of toll-like receptor 7 (*citlr7*) gene [213] and one SNP in an unknown gene *C7N1 *[214] in grass carp *Ctenopharyngodon idella*. In golden pompano (*Trachinotus ovatus*) from China, out of over 700,000 SNPs surveyed, four were significantly associated with hypoxia tolerance [215]. A correlation of 47 SNPs with a yearly mean water temperature has been reported for tarakihi fish (*Nemadactylus macropterus*) from around New Zealand and Tasmania [66].

SNP alleles associated with the warm temperature of the sea floor were identified in the giant California sea cucumber (*Parastichopus californicus*) [216]. American lobster (*Homarus americanus*) populations are under fishery pressure and have weak genetic structure on the Atlantic coast of North America as shown with neutral markers. In an extensive survey of over 14,000 SNPs, putatively adaptive SNPs located in such genes as a thermal stress response, salinity tolerance and growth demonstrated a higher sensitivity for finding a fine-scale population genetic structure and the capabilities of the identification of management units [217]. European green crab *Carcinus maenas* is invasive on the Pacific coasts of North America and has been found to have some SNPs linked to a chromosomal inversion associated with latitude and winter temperature [218]. In the studies of myosin heavy-chain proteins important for the muscle growth gene *MYH* in *Pinctada fucata martensii* had 18 out of 95 SNPs discovered in the exons region, were significantly associated with growth, of which 2 were confirmed in 2 populations [219]. In the studies of newly established populations of the marine snail *Nerita yoldii* north of the Yangtze River estuary, neutral SNPs confirmed that it consisted of founders and was distinct from the southern source population [220]. The new marginal population went through a bottleneck effect. The analysis of outlier SNPs showed a higher level of heterozygosity in comparison with the source populations. The expansion of populations north of the Yangtze River estuary can be explained by the building of coastal constructions, which is a hard substrate favorable for this species of snails and a warming climate. A new population was characterized by different transcriptomic and physiological reactions to the changes of temperature, which can be considered as an adaptation to newly colonized environment.

## 7. The Genetic Effects of Size-Selective Fisheries

Since the late 1990s, there have been publications indicating that fishing can be a selection factor inducing genetic changes and evolution in the exploited populations. Experimental studies of the impact of fishing on genetic changes in fish populations were carried out on several species of fish [221]. Common garden experiments and research combined with harvesting (fishing and angling) were performed in small ponds on largemouth bass (*Micropterus salmoides*), in natural conditions such as small lakes (trout *Oncorhynchus mykiss*), or fish (guppy *Poecilia reticulata*) transferred in creeks between environments with different pressures of predators isolated by waterfalls. It has been shown that fishing selection can have similar effects to the pressure of natural predators. The effects observed were changes in the time to reaching maturity for reproduction, size and growth rate, rapid changes in phenotypic features and life cycles [221]. The experimental size-selective catches of captive tanks populations of *Danio rerio* originated from natural conditions and caused evolutionary changes in life cycles in the 5th generation: increased energy allocation for reproduction, reduced adult size (breeding maturity) and less exploratory behavior and boldness [222]. The observed changes increased the population growth but decreased the population recovery. The populations have adapted to harvesting large individuals, but have reduced their ability to tolerate natural selection in favor of larger individuals. Harvest-induced genetic changes were found using 371 SNPs, including 34 outliers located in functionally important genes. The analysis of a large number of SNPs (over 21,000) confirmed this differentiation and showed that many SNPs are located in differentially expressed genes [222]. Also under experimental conditions, catches of *Danio rerio* caused a phenotypic selection in growth, metabolism and social behavior [223]. A genomic analysis of 5.6 million mapped SNPs suggested a selective effect of trawling. The comparison of captured and surviving fish revealed 480 outlier SNPs, which confirmed the genetic differences between these samples and the selection at the genome level.

Six captive populations of the marine Atlantic silverside fish, *Menidia menidia*, were created in tanks of 1100 individuals each [224]. Two populations were harvested for the largest 90% of fish, two populations for the smallest 90% of fish and two control populations were harvested randomly. After four generations, the first group decreased in weight, whereas the second group substantially increased in weight and gamete production. The authors explained their result by genetic changes in somatic growth. Genomic changes in these same and natural populations were surveyed with 2.36 million SNPs [225]. A reduction in the genetic diversity (polymorphic sites and nucleotide diversity) was observed in all captive groups, but it was higher in the harvested than in control groups. The highest geographic structure (northern—southern populations) in natural populations was found for 357 SNPs. In the north, the short summer favors the alleles associated with fast growth, most of which was more frequent in the group with the smallest fish removed (up selected), with a reversed principle for slow-growing group. On the basis of the presented research, it could be concluded that size-selective fishing can lead to genetic and phenotypic changes in natural populations, which make it difficult, and probably impossible, to recover a population (return it to the original structure of the population size and abundance) after reducing fishing intensity. This may mean that in the event of unfavorable changes in environmental conditions, the population will not be able to recover due to permanent changes caused by intensive fishing. However, the results of experimental studies cannot be directly related to the populations living in natural conditions for several reasons, such as the variability of environmental and ecological factors, biological and genetic diversity of natural populations, large effective population sizes and the duration of the fishing pressure.

Using approximately 9000 SNPs, changes in the structure of harvested populations, neutral diversity and signatures of selection have been reported in Mistassini Lake, Quebec for 15 years, whereas no such changes have occurred in the reference, weakly exploited population of walleyes, *Sander vitreus* [226]. Populations of this same species in Alberta, Canada were exposed to intensive sport fishery, which caused collapses of populations in a third of water bodies [227], and the recovery plan was implemented in 1996 including minimum size, and limits in catches in these waters. Archival samples from 1973 and samples from six lakes collected in 2000–2005 were genotyped with SNPs by sequencing. Assignment decline in genetic diversity have been observed in the walleye population in Smoke Lake from 1973 to 2000, in comparison with samples collected before and after the population collapse (428 SNPs). However, the authors did not exclude the possibility that this population could have been supplemented with stocking material, which could have caused the reduction in its genetic diversity. In the lake whitefish, *Coregonus clupeaformis*, the Lesser Slave Lake population experienced a collapse due to overfishing and in 1965–1972 a ban on fishing was imposed. Out of 61 SNPs located in the genes, one was identified as a candidate SNP under divergent selection, which may be explained as an effect of overfishing [9]. According to [228], the gillnet fishing of fast-growing domesticated ‘Californian’ genotypes of rainbow trout *O. mykiss* was more intensive in comparison with slow-growing wild genotypes in two small lakes stocked for research purposes in southwestern British Columbia, Canada. However, no changes in the genetic composition as analyzed with 349 neutral SNPs have been observed, despite the collapse of the population of the European anchovy, *Engraulis encrasicolus* in the Biscay Bay in the period 2002–2010 [229]. A comparison of cod *Gadus morhua* archival samples collected in 1907 (Lofoten Island, Norway) and 1940 (Newfoundland, Canada) and modern samples in 2013 (Canada) and 2011 and 2014 (Norway) from these same locations using 346,290 SNPs obtained by whole-genome sequencing did not reveal differences in the genetic diversity in populations from before and after periods of overfishing and a rapid decline of the age of maturation [230]. No overfishing effect has been found due to a lack of evidence of a loss of genetic diversity in albacore tuna *Thunnus alalunga*, populations over time [231]. Hutchings and Kuparinen [232] in their literature review concluded that fishery-induced changes (evolution) at the genome level in natural populations are difficult to find and possibly are not very important in comparison with other factors (environmental and ecological). Negative measurable effects on populations that affect genetic polymorphisms, can be expected from a reduction in the effective population size below 100 individuals, which is very rarely the case in marine environments and is more frequent in fragmented freshwater creeks, ponds and periodic rivers. Fishery-induced selection against fast growing and bold fish could be observed in wild populations if QTLs and SNPs located in the candidate genes are identified. In the case of polygenic traits, the fishery-induced evolution may not be detectable in wild populations.

## 8. Conclusions

There is growing awareness that local populations as well as genetic structures on a large scale in marine and freshwater environments should be protected, along with the ecosystems to which they belong. An increasing number of aquatic species has been studied for genetic polymorphisms, which extends the knowledge on their natural populations. In previous years, population polymorphism studies were carried out using various genetic markers, some of which turned out to be particularly useful and enabled obtaining results on a local and macro-geographical scale comparable in different laboratories, e.g., allozymes, mtDNA, single nuclear DNA loci, restriction fragment length polymorphism (RFLP) and microsatellites. In the last decade, thanks to the progress of genomics and next-generation sequencing (NGS) methods, the use of single nucleotide polymorphism (SNP) markers has become particularly popular in population genetics studies. Although the reduction in the cost of sequencing whole genomes makes it possible to use it in population studies, it does not yet apply to non-model species. It allows the discovery of millions of SNPs, from which a smaller or larger number (e.g., more than 10,000) can be selected for population genetics research. However, with such a large number of SNPs, a subset of diagnostic SNPs can be used for cost-effective genotyping in individual laboratories. It should be noted that the use of these revolutionary markers in population genetic studies confirmed the results obtained earlier with other markers, e.g., allozymes or microsatellites, and substantially extended them. The advantage of using SNPs lies not only in the quantity, but also in the possibility of identifying neutral loci showing differentiation as well as loci under the action of selection. Moreover, the use of genetic mapping enables the discovery of SNPs associated with candidate genes localized in quantitative trait loci (QTLs) of interest and finding favorable genotypes in natural populations. This could be helpful in the application of gene-editing technology for aquaculture purposes.

The most successful application of SNPs in population genetics lies in their ability to identify populations on a local and intercontinental scale, which is of fundamental importance for conservation and management. SNPs can be used for the precise identification of the origin of introduced populations, finding interactions between natural and introduced or invasive populations and species, identification of hybrids, levels of inbreeding and estimating effective population sizes with the linkage disequilibrium method. Enhancement leads to a reduction in the diversity of the population compared to other wild populations because the number of broodfish contributing to stocking material production is usually too low. Domesticated broodfish should be replaced with individuals originating from wild populations.

SNP genetic data can be used for finding the adaptations of populations to changing environmental factors. Neutral and outlier SNP alleles can be correlated with the environmental variables and show usually weak linkages. The genotyping of a larger number of SNPs located in genes with known function can reveal adaptations to the specific environmental drivers, such as temperature, salinity, oxygen, pH, precipitation, etc. Such knowledge will enhance the power to predict the ability of populations to adapt to expected climate changes and help to implement more efficient conservation prioritization. Studies conducted on natural populations and stocks in aquaculture enabled the identification of QTLs, including SNPs located in genes, which regulate quantitative characters such as growth, age and size of fish at maturity, resistance to pathogens, stress and tolerance to varied environmental conditions. Fish stocks subjected to strong size-selective fishery pressure under experimental or semi-natural culture conditions change their performance and experience alteration in genetic composition (allele frequencies) over several generations depending on the individual size. The negative measurable effects on populations affecting genetic polymorphism can be expected from a reduction in the effective population size below 100 individuals, which is very rarely the case in marine environments and is more frequent in fragmented freshwater creeks, ponds and periodic rivers. Fishery-induced selection against fast-growing and bold fish could be observed in wild populations if QTLs and SNPs located in the candidate genes are identified. In the case of polygenic traits, the fishery-induced evolution may not be detectable in wild populations.

It is postulated that protection measures should encompass not only pristine endangered or overfished natural populations, but also non-endangered species populations from unnecessarily released hatchery animals. In the case of the foreseen extinction of local populations, supportive breeding and enhancement of populations can be considered as an ultimate measure for rescuing declining populations. For the purposes of population analyses, it is imperative that the most appropriate set of baseline and reference populations or groups of individuals be chosen. Otherwise, the low number of loci used in an economical survey can determine inappropriate and misleading results, as an occurrence or lack of temporal changes in a population, or the rejection or confirmation of the presence of a taxon in a geographic region under study. The full potential of SNPs in studies of populations has yet to be explored in population genomic research.

## Figures and Tables

**Table 1:** List of species with sets of SNPs reported for the genetic structure studies of wild populations.

Common Name	Species	Region/Country	Reference
Chum salmon	*Oncorhynchus keta*	North Pacific, Alaska, Japan, North America, Asia	[25,26]
Rainbow trout, steelhead	*Oncorhynchus mykiss*	California, USA, North America	[27]
Rainbow trout, steelhead	*Oncorhynchus mykiss*	Pacific coast and hatcheries, USA, North America	[28]
Chinook salmon	*Oncorhynchus tshawytscha*	Pacific coast, USA, Canada, North America	[29,30]
Coho salmon	*Oncorhynchus kisutch*	Pacific coast of Russia and North America, Asia	[31]
Atlantic salmon	*Salmo salar*	North Atlantic, Europe, North America	[32,33]
Brown trout	*Salmo trutta*	Europe	[34,35,36]
Brook charr	*Salvelinus fontinalis*	Quebec, Canada, North America	[37]
Arctic charr	*Salvelinus alpinus*	Labrador, Canada, North America	[38,39]
Sichuan taimen	*Hucho bleekeri*	Taibai River, Yangtze River, China, Asia	[40]
European sea bass	*Dicentrarchus labrax*	Adriatic clade, Italy, Europe	[41]
Striped bass	*Morone saxatilis*	Coast of USA, North America	[42]
White bass	*Morone chrysops*	Coast of USA, North America	[42]
Atlantic herring	*Clupea harengus*	North Sea, Scandinavia and Baltic, Europe	[43,44]
European anchovy	*Engraulis encrasicolus*	Ibera, Spain, Europe	[45]
Atlantic cod	*Gadus morhua*	North Atlantic, North America, Europe	[46,47,48,49]
Pacific cod	*Gadus macrocephalus*	Korean Peninsula coast, South Korea, Asia	[50]
Gilthead sea bream	*Sparus auratus*	Faro, Portugal, Europe	[51]
Gilthead sea bream	*Sparus aurata*	Atlantic Iberia and Mediterranean Sea, Europe	[52]
Sea bass	*Dicentrarchus labrax*	Faro, Portugal, Europe	[51]
European seabass	*Dicentrarchus labrax*	Mediterranean Sea, Europe	[52]
Turbot	*Scophthalmus maximus*	Cantabria, Spain, Europe	[53]
Tongue sola	*Cynoglossus semilaevis*	Bohai and Yellow seas, China, Asia	[54]
Yellowtail kingfish	*Seriola lalandi*	Bohai, China, Great Australian Bight	[55]
Miiuy croaker	*Miichthys miiuy*	Zhoushan Fisheries Research Institute, Zhejiang, China	[56]
Lined seahorse	*Hippocampus erectus*	West Atlantic, Zhanjiang Seahorse Center, China, Asia	[57]
Nile tilapia	*Oreochromis niloticus*,	Shanghai aquaculture, Singapore, Asia	[58]
Mozambique tilapia	*O. mossambicus*,	South Africa—Singapore aquaculture, Singapore	[58]
Yellow catfish	*Pelteobagrus fulvidraco*	Hubei province, China, Asia	[59]
Indian catfish	*Clarias batrachus*	Indian subcontinent, India, Asia	[60]
Blunt snout bream	*Megalobrama amblycephala*	Yangtze River, China, Asia	[61]
Hoki	*Macruronus novaezelandiae*	New Zealand, Tasmania, Australia	[62]
Tambaqui	*Colossoma macropomum*	Amazon River, Brazil, South America	[63]
Pacu	*Piaractus mesopotamicus*	Paraná River, La Plata Basin, South America	[64]
Walleye	*Sander vitreus*	Lake Winnipeg, Manitoba, Canada, North America	[65]
Tarakihi	*Nemadactylus macropterus*	Tasmania, New Zealand, Australia	[66]
Surgeonfish	*Acanthurus triostegus*	Hawaiian Islands, USA, North Pacific	[67]
Ocellated icefish	*Chionodraco rastrospinosus*	Antarctic Peninsula and islands, Antarctic	[68]
Hammerhead shark	*Sphyrna lewini*	Indo-Pacific, Australia, Asia, North America	[69]
Blue skate	*Dipturus batis*	North-East Atlantic, Great Britain, Europe	[70]
Pouched lamprey	*Geotria australis*	New Zealand	[71]
Sea cucumber	*Apostichopus japonicus*	Yellow Sea NW coasts, China, Asia	[72]
Pacific white shrimp	*Litopenaeus vannamei*	Shenzhen coast, introduced, China, Asia	[73]
Black tiger shrimp	*Penaeus monodon*	Indo-Pacific, Malaysia, Japan, Asia	[74]
European lobster	*Homarus gammarus*	costal European waters, Europe	[75]
Swimming crab	*Portunus trituberculatus*	Bohai, Yellow and East China seas, China, Asia	[76]
Blue swimming crab	*Portunus pelagicus*	China, Asia	[77]
Common whelk	*Buccinum undatum*	Southern Great Britain, United Kingdom, Europe	[78]
Abalone	*Haliotis midae*	Roman Bay Sea Farm, Gansbaai, South Africa, Africa	[79]
Freshwater pearl mussel	*Hyriopsis cumingii*	Poyang Lake, Duchang, Jiangxi Province, China, Asia	[80]
Blood clam	*Tegillarca granosa*	Xiangshan, Zhejiang Province, China, Asia	[81]
Pacific oyster	*Crassostrea gigas*	Yellow Sea, NW coasts, China, Asia	[82]
Pacific oyster	*Crassostrea gigas*	SW Europe, introduced, Europe	[83]
Estuarine oyster	*Crassostrea ariakensis*	Coast, China, Asia	[84]
European flat oyster	*Ostrea edulis*	SW Europe, native Europe	[83]
Silver-lipped pearl oyster	*Pinctada maxima*	Islands: from Raja Ampat to Bali, Indonesia, Asia	[85]
Razor clam	*Sinonovacula constricta*	Ningbo City, Zhejiang Province, China, Asia	[86]
Northern bay scallop	*Argopecten i. irradians*	Yellow Sea, NW coast, introduced, China, Asia	[87]
Southern bay scallop	*A. i. concentricus*	Zhanjiang, Guangdong Province, China, Asia	[87]
Southern bay scallop	*A. i. concentricus*	Shenzhen, Guangdong Province, China, Asia	[88]
Zhikong scallop	*Chlamys farreri*	Yellow Sea, NW coast, China, Asia	[89]
Surf clam	*Mesodesma donacium*	Caleta San Pedro, Coquimbo, Chile, South America	[90]
Blue mussels	*Mytilus* spp.	Mediterranean, North Atlantic, North America, Europe	[91,92]
Coral	*Acropora digitifera*	Nansei Islands, Japan, Asia	[93]
Table coral	*Acropora downingi*	Oman, Qatar, United Arab Emirates, Oman, Middle East	[94]
Corals	*Porites* sp., *Pocillopora acuta*	Singapore Straits, Singapore, Asia	[95]

## Data Availability

Not applicable.
